# Some Cancer Studies

**Published:** 1906-08-11

**Authors:** 


### Some Cancer Studies.

Among the numerous and difficult questions presented to the medical profession by the phenomena of disease, the problems associated with the existence of cancerous or malignant growths stand in the first rank. The mystery which surrounds these problems is all the more remarkable in view of the frequency with which such growths occur. Here is no rare and peculiar condition coming only at occasional instances under the observation of a few favoured observers. On the contrary, cancer as a clinical fact is a relatively frequent event, and its manifestations are constantly exhibiting themselves for the observation of every practitioner. The disease is common, distressingly common indeed, and from it arises a long and melancholy tale of human misery and suffering. Yet in spite of what seems abundance of opportunity for observation, neither the stimulus of scientific curiosity nor the promptings of human sympathy have led to the discovery of the cause and essential nature of cancerous disease. The secret, so far, defies detection. It is impossible to imagine a more graphic illustration of the extraordinary difficulty and intricacy of many of the questions with which the medical profession is confronted. The illustration becomes all the more vivid when it is remembered that during recent years the most thorough and advanced methods of research have been systematically applied to the solution of the cancer problem. Yet the complex conditions which surround and influence the subtle and mysterious processes of tissue growth and vital chemistry form a barrier which human ingenuity and industry have not yet been able to surmount.

But whilst facts compel an admission that the mystery of cancer remains unsolved, there are reasons to anticipate that the difficulties will not permanently defy human resolution. One such reason is certainly to be found in the organised scientific quest that is at present being prosecuted by such agencies as the Imperial Cancer Research Fund and the Cancer Research Laboratories of the Middlesex Hospital. To those who have confidence in the patient processes of scientific observation and experiment it is impossible to read the reports issued by these institutions without the production of sanguine anticipations. Here, it is obvious, skilled observers are coming into immediate and critical contact with the ultimate facts of cancer, and, remembering former triumphs, no one but a confirmed pessimist can doubt that sooner or later victory will be assured To a public impatient of results many of the published records may seem to have little practical value and to be remote from the main purpose—namely, the cure of patients. But the medical profession will not so read the immediate issue of long and laborious work. Mere clinical facts observed for years, and even for centuries, left the problem of cancer a hopeless mystery. As a contrast, the adoption of laboratory and experimental methods has within a few years led to the discovery of a large body of new facts, many of which, whilst important in themselves, are still more important in relation to the lines of inquiry which they suggest. Is it not, for example, highly significant that it is now possible to reproduce in mice and at will all the features of spontaneous cancer, and to protect healthy mice from all the consequences of inoculating them with experimental cancer? Between these facts and the prevention and cure of malignant disease in human beings there is doubtless an enormous gap. But the step in advance is a distinct one, and it carries hope and encouragement. Like other gains due to the application of scientific method it remains a permanent contribution to knowledge, and from it as a starting-point new developments are certain to take origin. Again, the reports of the minute and searching study of cases of cancer conducted in the wards and laboratories of the Middlesex Hospital, though hardly affording any broad positive result capable of collection in a general statement, are a guarantee that no aspect of the question is being neglected. Here, too, it must not be forgotten that negative conclusions are not without value. They at least help to exclude suggestions which have enjoyed a more or less authoritative recognition, and so tend to keep the research movement towards helpful and promising directions.

There is one negative issue of the cancer inquiry which many will read with regret. It is the proved uselessness of a number of substances which have been more or less urgently vaunted as "cures." Thus at the Middlesex Hospital violet leaves, betel leaves, uranium salicylate, caustic paste, and a liquid preparation of radium have all been systematically tested. In no single instance, however, was any benefit observed, and practically the same conclusions are recorded by the Imperial Cancer Research Fund. It is hardly by some happy guess or lucky chance that a medicinal agent for the cure of cancer will be discovered. But there is reason to expect that the prosecution of the scientific studies now in effective operation will in time secure the control of this terrible and widespread disease.

